# Expression profiles of long noncoding RNAs and mRNAs in peripheral blood mononuclear cells of patients with acute myocardial infarction

**DOI:** 10.1097/MD.0000000000012604

**Published:** 2018-10-12

**Authors:** Pingsen Zhao, Heming Wu, Zhixiong Zhong, Qifeng Zhang, Wei Zhong, Bin Li, Cunren Li, Zhidong Liu, Min Yang

**Affiliations:** a Clinical Core Laboratory; b Center for Precision Medicine; c Center for Cardiovascular Diseases, Meizhou People's Hospital (Huangtang Hospital), Meizhou Hospital Affiliated to Sun Yat-sen University; d Guangdong Provincial Engineering and Technology Research Center for Molecular Diagnostics of Cardiovascular Diseases; e Meizhou Municipal Engineering and Technology Research Center for Molecular Diagnostics of Cardiovascular Diseases; f Meizhou Municipal Engineering and Technology Research Center for Molecular Diagnostics of Major Genetic Disorders, Meizhou, PR China.

**Keywords:** acute myocardial infarction, bioinformatics analyses, LncRNA, peripheral blood mononuclear cells

## Abstract

Acute myocardial infarction (AMI) is the most serious type of coronary atherosclerotic diseases. The incidence of AMI in some countries increases year by year, and shows younger trend. Some study indicated that abnormal expression of lncRNAs was closely related to cardiovascular disease. The aim of this study was to examine the lncRNA expression profiles in peripheral blood mononuclear cells (PBMCs) of patients with AMI through controlled studies.

In the present study, we examined the lncRNA and mRNA expression profiles in 8 patients with AMI, with 7 NCA (noncoronary artery) subjects as controls using RNA sequencing protocol (RNA-seq) on the Illumina HiSeq 4000 platform. The differentially expressed lncRNAs were selected for bioinformatic analysis including gene ontology (GO) enrichment and Kyoto Encyclopedia of Genes and Genomes pathway (KEGG). Quantitative real time PCR (qRT-PCR) was used to confirm the differential expression of lncRNAs.

We kept about 11.29 gigabase (Gb) high-quality sequence data while the Q30 ranged from 94.39% to 95.19% for each sample. Compared to the lncRNA expression profiles of NCA controls, a total of 106 differentially expressed lncRNAs were discriminated in AMI patients, including 40 upregulated lncRNAs and 66 downregulated lncRNAs (*P < *.05). Among the genes corresponding to the identified mRNAs, 2905 genes are involved in biological processes, 339 in cellular components, and 501 in molecular functions. Based on the KEGG pathway analysis, the most enriched pathways corresponding to the differentially expressed lncRNAs were associated with systemic lupus erythematosus, alcoholism, oxidative phosphorylation, Parkinson's disease and viral carcinogenesis, and so on. Further, 3 upregulated and 3 downregulated lncRNAs were randomly selected for qRT-PCR verification and the results of qRT-PCR were consistent with the findings obtained from RNA sequencing analysis.

As a result, differential expression profiles of lncRNAs in AMI were identified in our study. The results suggested that lncRNAs may play important roles in the biological and pathological processes of AMI. These findings may provide useful reference for the early diagnosis and risk stratification of AMI patients. To enlarge the sample size in the next step will be needed for further research to confirm our results.

## Abstract 2

Supplemental Digital Content is available in the text

## 1 Introduction

Cardiovascular disease is one of the major diseases that threaten human health. Acute coronary syndrome (ACS) is a group of clinical syndromes characterized by rupture or invasion of coronary atherosclerotic plaques secondary to complete or incomplete occlusive thrombosis, including ST segment elevation myocardial infarction (STEMI), non-ST segment elevation myocardial infarction (NSTEMI) and unstable angina (UA). STEMI and NSTEMI are collectively referred to as acute myocardial infarction (AMI).^[1,2]^

AMI has the characteristics of rapid onset, rapid course and high mortality. It is the most serious coronary atherosclerotic disease and the main cause of death from nononcological diseases in some countries. In recent years, the incidence of AMI in some countries rises year by year, and shows younger trend.^[3,4]^ At present, the pathogenesis of cardiovascular disease has not yet been fully elucidated. Abnormal expression of inflammatory, protease, and apoptotic molecules was associated with damage to cardiomyocytes and cardiovascular disease.^[5]^ It is well known that many of the risk factors leading to cardiovascular disease have been identified, including age, gender, smoking, alcohol abuse and comorbidities such as diabetes, dyslipidemia, arterial hypertension, and peripheral vascular disease.^[6]^ Despite the high risk of AMI, we lack the effective predictive diagnosis.^[7–9]^

The occurrence and development of cardiovascular disease include the occurrence of vascular wall inflammation, vascular injury and plaque formation, which involve the release of molecules in the immune system.^[10,11]^ A peripheral blood mononuclear cell (PBMC) is any peripheral blood cell having a round nucleus.^[12]^ PBMCs include lymphocytes (T cells, B cells, and NK cells), monocytes, and dendritic cells. In humans, the frequencies of these populations vary across individuals. These cells can be further classified into various functional subtypes based on the expression profiles of specific cytokines, surface markers, or transcription factors. Human immune system studies rely heavily on the phenotypic and functional assessments of PBMCs. In order to take advantage of PBMCs for human immune studies, it is important to know what populations are represented in peripheral blood and how PBMC populations differ in distribution and function from tissue immune cells. Also it is critical to become familiar with the identifying surface and intracellular markers and the types of assays best suited for human PBMC studies.^[13–15]^ So the expression profiles in peripheral blood mononuclear cells can correlate with AMI progression.

Long noncoding RNAs (lncRNAs) are noncoding RNAs (ncRNAs) with a transcript length of 200 nt and without protein coding function. lncRNAs were initially considered as the “noise” of genome transcription. Recent studies have shown that lncRNAs are closely related to X chromosome silencing, genomic imprinting, chromatin modification, transcriptional activation, transcriptional interference, and nuclear transport.^[16–20]^ It engages in the regulation of the growth and development of the individual, the differentiation, proliferation, apoptosis of cells, as well as other life activities. Although the specific functions of lncRNAs have not been clarified, the studies have shown that the abnormal expression of lncRNAs were highly correlated to cardiovascular diseases.^[21–23]^

The aim of this study was to examine the lncRNAs expression profiles in peripheral blood mononuclear cells (PBMCs) of patients with AMI through controlled studies. According to the comparison of the lncRNAs expression profiles among 15 subjects, we desire to obtain a correlation between lncRNAs and AMI. These results will provide a useful reference for further exploration of the role of lncRNAs in the progression of AMI.

## 2 Materials and methods

### 2.1 Subjects

AMI was diagnosed by coronary angiography, dynamic evolution of electrocardiogram and dynamic changes of serum markers. The patients with ST segment elevation were diagnosed with ST segment elevation myocardial infarction, and those without ST segment elevation were diagnosed as non-ST segment elevation myocardial infarction. 15 subjects visited Meizhou People's Hospital located Guangdong province of China through February 2016 to April 2017 involved in this study, including 8 males and 7 females and aging from 43 to 68 years. Around 15 subjects were classified into 2 groups: NCA (noncoronary artery, 7 subjects) and AMI group (8 subjects). This study was performed in accordance with the Declaration of Helsinki, and was supported by the Ethics Committee of the Meizhou People's Hospital.

### 2.2 Samples collection and total RNA extraction

Around 3 mL of blood samples for the measurement of lipid levels were obtained from each subjects, plasma was separated and stored at −80 °C till further analysis. Plasma levels of triglyceride (TG), total cholesterol (TC), high-density lipoprotein (HDL), and low-density lipoprotein (LDL) were measured.

Whole blood samples (6 mL from peripheral venous blood) were collected from patients with AMI at the onset of symptoms and NCA controls. Blood samples were taken from antecubital vein and stored in vacuum tubes containing ethylenediaminetatraacetic acid (EDTA), on the upside down gently mix 10 times, immediately saved the blood in 4 °C. Plasma should be separated within one hour, and were centrifuged at 1500 r/min centrifugal 10 minutes to get the upper plasma samples, transferred the plasma to 1.5 mL RNA (RNasefree) centrifuge tube for extraction of RNA, packed stored at −80 °C.

Total RNA was extracted from the plasma using TRIzol reagent (Invitrogen, Carlsbad, CA, USA) according to the manufacturer's instructions. The quantity and purity of total RNA were evaluated by Nanodrop 2000, and the RNA Nano 6000 Assay Kit of the Agilent Bioanalyzer 2100 system (Agilent Technologies, CA) was used to analyze RNA integrity.

### 2.3 Preparation for lncRNA sequencing library

A total amount of 3 μg RNA was utilized in the RNA sample preparations, strictly according to the manufacturer's protocol. Firstly, ribosomal RNA was removed by using an Epicentre Ribo-zeroTM rRNA Removal Kit (Epicentre, Madison, WI), and residual RNAs were cleaned up using ethanol precipitation. Sequencing libraries were generated using the rRNA-depleted RNA with NEBNextUltraTM Directional RNA Library Prep Kit for Illumina (NEB). The RNA integrity was evaluated by using the RNA Nano 6000 Assay Kit of the Bioanalyser 2100 system (Agilent Technologies, CA). The libraries were sequenced on an Illumina Hiseq 4000 platform according to the commercially available protocols and 150 bp paired-end reads per sample were generated.

### 2.4 High throughput sequencing

Sequencing libraries were generated using NEBNext Multiplex Small RNA Library Prep Set for Illumina (NEB, USA.) according to manufacturer's protocol. After the qualification of the library, the different libraries were sequenced in accordance with the requirement of the effective concentration and the amount of data of the machine under the target pooling, and then library sequencing was carried out on Illumina HiSeq 4000 platform according to the commercially available protocols in ShenZhen Realomics Inc.

### 2.5 Identification of differently expressed genes

The analysis of differences in lncRNA expression of 2 groups samples was performed using the DEGseq (2010) R package. *P*-value was adjusted using *q*-value. *q*-value < 0.05 and |log2(foldchange)|>1 were set as the threshold for significantly differential expression by default.

### 2.6 Quantitative real-time polymerase chain reaction (qRT-PCR)

To validate the reliability of RNA sequencing data, differentially expressed lncRNAs were randomly selected and qRT-PCR was employed to examine the expression level of lncRNAs. Total RNA were extracted from the PBMCs using TRIzol reagent (Invitrogen, Carlsbad, CA) according to the manufacturer's instructions. And qRT-PCR reactions were performed by using Luna Universal One-Step RT-qPCR kits (New England Biolabs, MA). The PCR reactions were carried out by the conditions: 15 s at 55 °C and 1 minute at 95 °C, followed with 40 cycles for 10 seconds at 95 °C and 30 seconds at 60 °C, and 30 seconds at 50 °C. Glyceraldehyde 3-phosphate dehydrogenase (GAPDH) was used as an internal control for measurement of lncRNAs and the relative expression levels of candidate lncRNAs were calculated using the 2^−ΔΔCT^ equation. At least triple experiments were subjected to qRT-PCR verification.

### 2.7 GO and KEGG enrichment analysis

The target mRNAs of lncRNAs were classified according to the principle of classification by Gene Ontology (http://www.geneontology.org/). GO gathers information from Gene Ontology and the NCBI database, annotates and classifies genes according to the biology process, molecular function and cellular location. KEGG (http://www.genome.jp/kegg/) is a comprehensive database for systematic analysis of gene function. It is based on the related knowledge of hand-painted metabolic pathways, mainly divided into categories: metabolism, genetic information processing, cellular processes, environmental information processing, organismal systems and human diseases. Each category is divided into some subitems.

### 2.8 Statistical analysis

**Figure 1:**
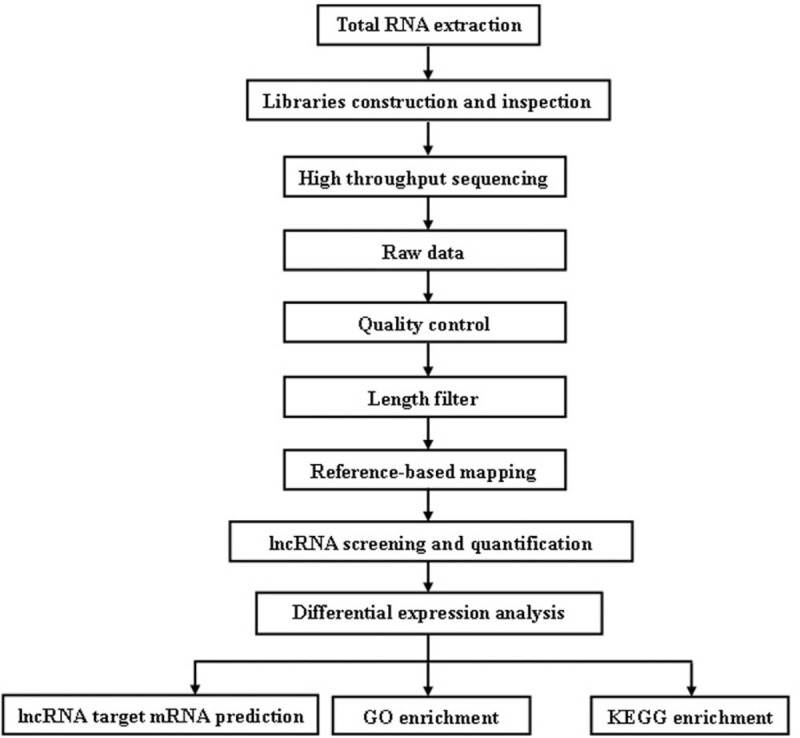
The workflow of the experiment.

SPSS statistical software version 19.0 was used for data analysis. Data were reported as the means ± SD. Chi-square and ANOVA tests were used to analyze the differences among the 2 groups. Statistical significance was set at a *P < *.05 (Fig. 1).

## 3 Results

### 3.1 The subjects’ clinical characteristics

The clinical characteristics of the 15 subjects in this study were presented in Table 1. There were higher systolic BP (*P = *.011) in the AMI patients than in NCA controls. There were no statistical differences in age, sex, smoking, drinking, diastolic BP, hypertension, diabetes, hyperlipidemia, total cholesterol (TC), triglycerides (TG), and high-density lipoprotein cholesterol (HDL-C) and low-density lipoprotein cholesterol (LDL-C) between the AMI patients and non-AMI controls.

**Table 1:**
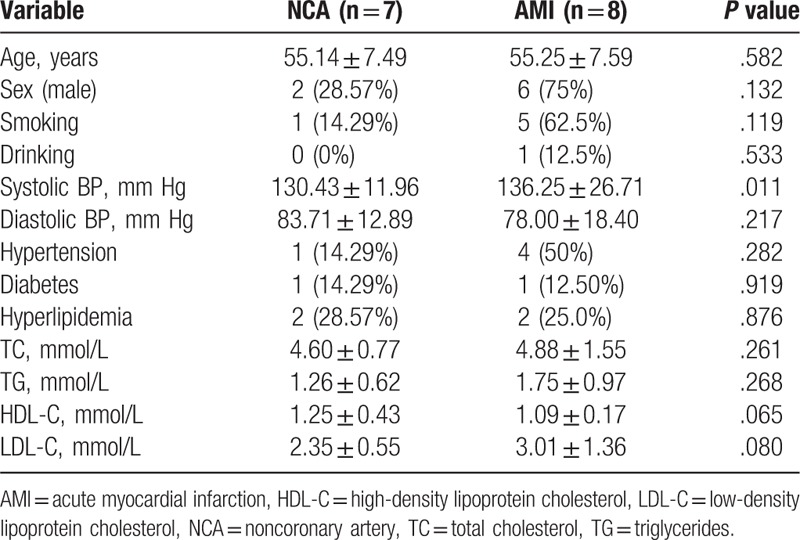
The baseline clinical characteristics.

### 3.2 Overview of lncRNA sequencing data

In this study, a total of 15 cDNA libraries were constructed and sequenced on the Illumina HiSeq 4000 platform by using total RNA from each sample. After quality control, we kept about 11.29 gigabase (Gb) high-quality sequence data while the Q30 ranged from 94.39% to 95.19% for each sample. Obviously, these results indicated that the quality of the 15 libraries were suitable for subsequent analysis. Details of data quality and data characteristics were listed in Supplementary Table S1 and Table S2.

### 3.3 Differentially expressed lncRNAs and mRNAs in PBMCs

**Figure 2:**
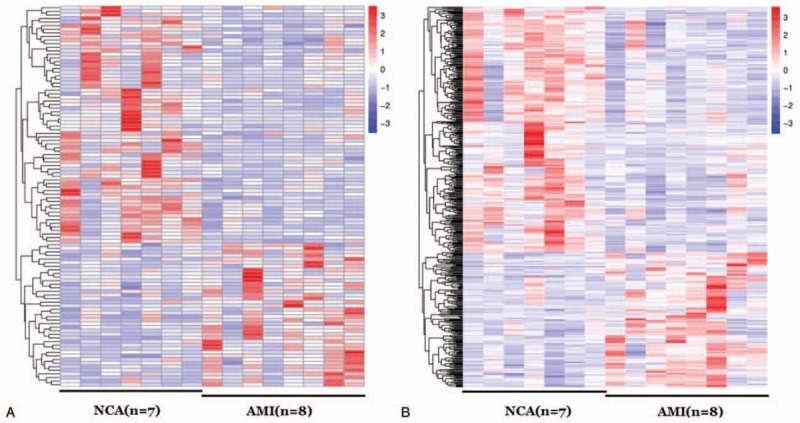
Hierarchical clustering of lncRNAs in AMI patients and NCA controls. The red and the green shades indicate an increase and a decrease in expression level, respectively, across all samples. (A) lncRNA; (B) mRNA. AMI = acute myocardial infarction, NCA = noncoronary artery.

To systematically investigate the expression levels of lncRNAs and mRNAs associated with AMI, lncRNA and mRNA sequence analyses were performed on the PBMCs of 8 AMI patients and 7 NCA controls. The results of hierarchical clustering showed the differential expression of lncRNAs (Fig. 2A) and mRNAs (Fig. 2B) between AMI patients and NCA controls. Expression values are represented in red and blue, indicating expression above and below the median expression value in each group. These observations suggested that potential changes between normal and AMI state were identified by differences in the expression profile of either lncRNAs or mRNAs related to AMI (Table 2).

**Table 2:**
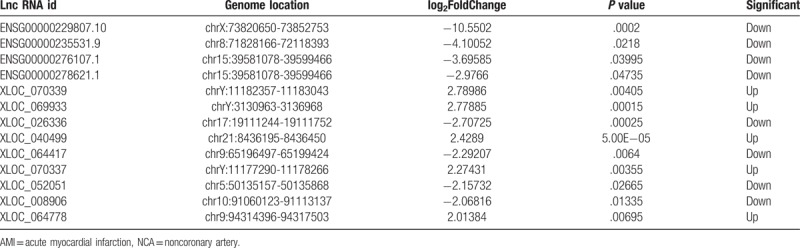
The most differentially expressed lncRNAs in AMI and NCA according |log2(foldchange)|>2.

**Figure 3:**
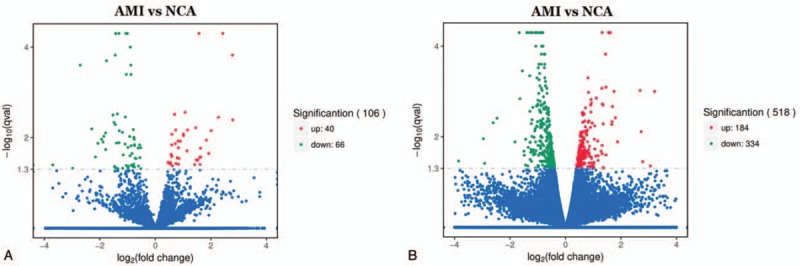
Volcano plot of differential lncRNA and mRNA expression. *X*-axis: log_2_(fold change); Y-axis: −1 × log_10_ (corrected *q*-value) for each probes. (A) AMI vs NCA in lncRNA; (B) AMI vs NCA in mRNA. AMI = acute myocardial infarction, NCA = noncoronary artery.

The volcano plots are presented as visualizations used to assess lncRNA and mRNA expressive variation between patients with AMI and NCA controls, respectively (Fig. 3). Compared to the lncRNA expression profiles of NCA controls, a total of 106 differential expression of lncRNAs were discriminated in AMI patients, including 40 upregulated lncRNAs and 66 downregulated lncRNAs (*P < *.05).

### 3.4 GO and KEGG pathway analyses

**Figure 4:**
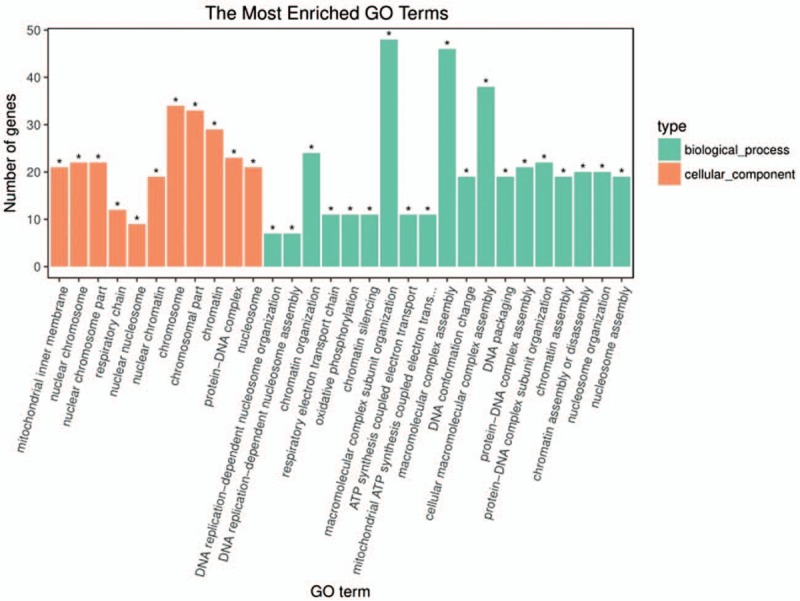
GO analysis of differentially expressed lncRNAs which covers 3 domains: biological process, cellular component and molecular function. *X*-axis: GO terms of biological process, cellular component and molecular function. The green column indicates biological process, the red column indicates cellular component and the blue column indicates molecular function. *Y*-axis on the left: numbers of genes (lncRNAs).

**Figure 5:**
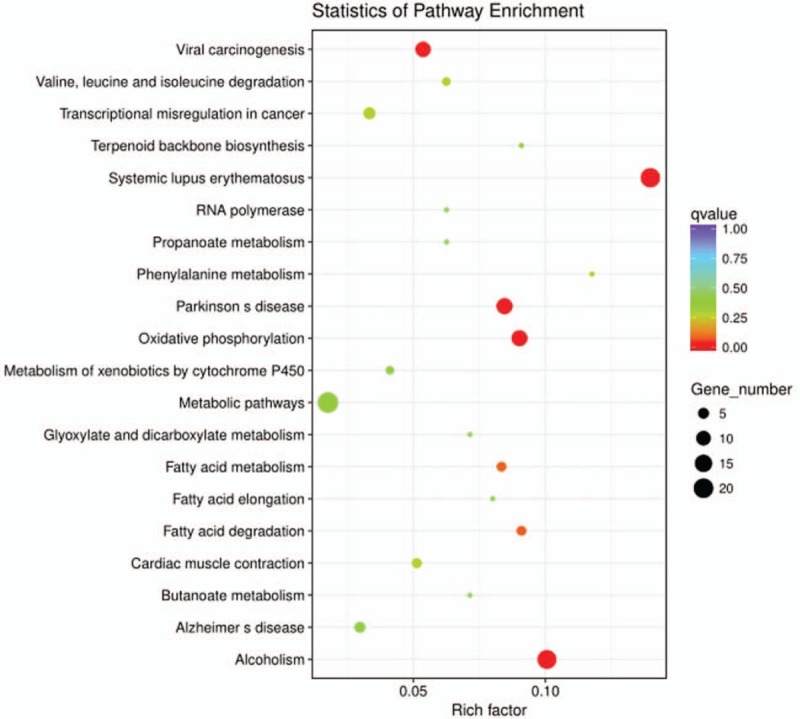
Pathway analysis of differentially expressed lncRNAs. Pathway analysis is a functional analysis mapping genes to KEGG pathway and other pathway databases. The lower the *P*-value, the more significant the pathway.

To further understand the function and related pathways of mRNA identified in this study, we performed GO and KEGG pathway analysis. Genes are classified according to biological processes, cell components and molecular functional tissues to reveal gene regulatory networks. Of the genes corresponding to the identified mRNA, 2905 genes are involved in biological processes, 339 in cellular components and 501 in molecular functions (Table 3  and Fig. 4). Based on the KEGG pathway analysis, we found that the most enriched pathways corresponding to the differentially expressed lncRNAs were associated with systemic lupus erythematosus, alcoholism, oxidative phosphorylation, Parkinson's disease and viral carcinogenesis, and so on (Table 4 and Fig. 5).

**Table 3:**
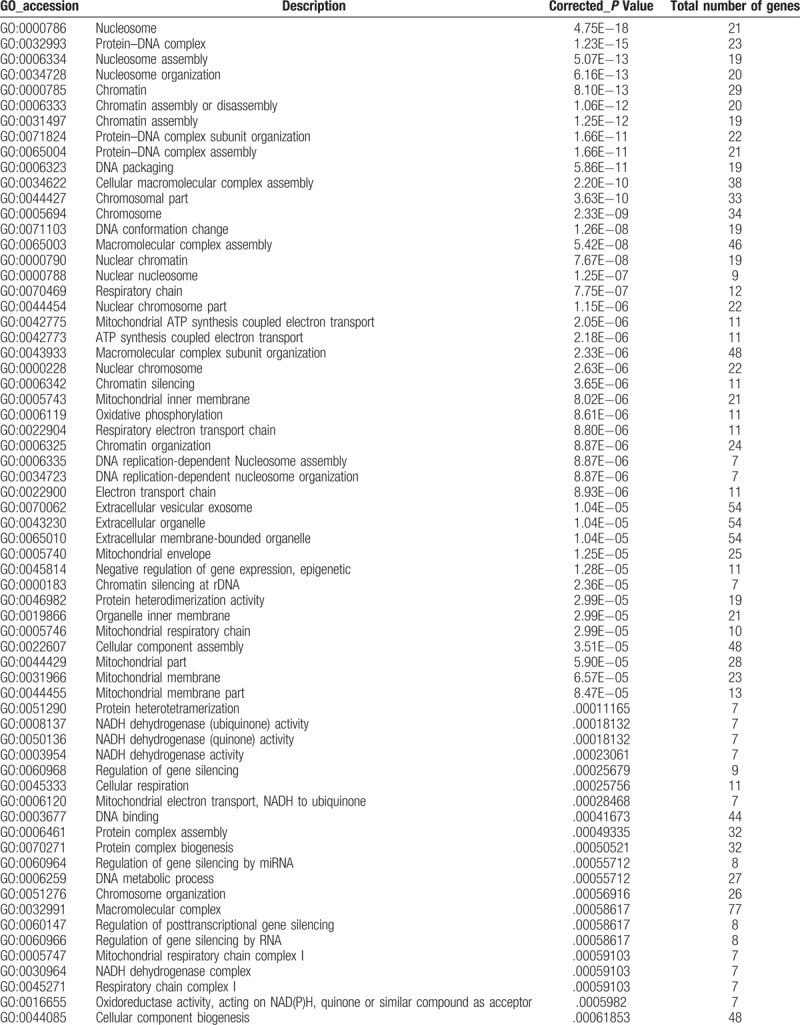
Significantly enriched gene ontology (GO) terms.

**Table 3 (Continued):**
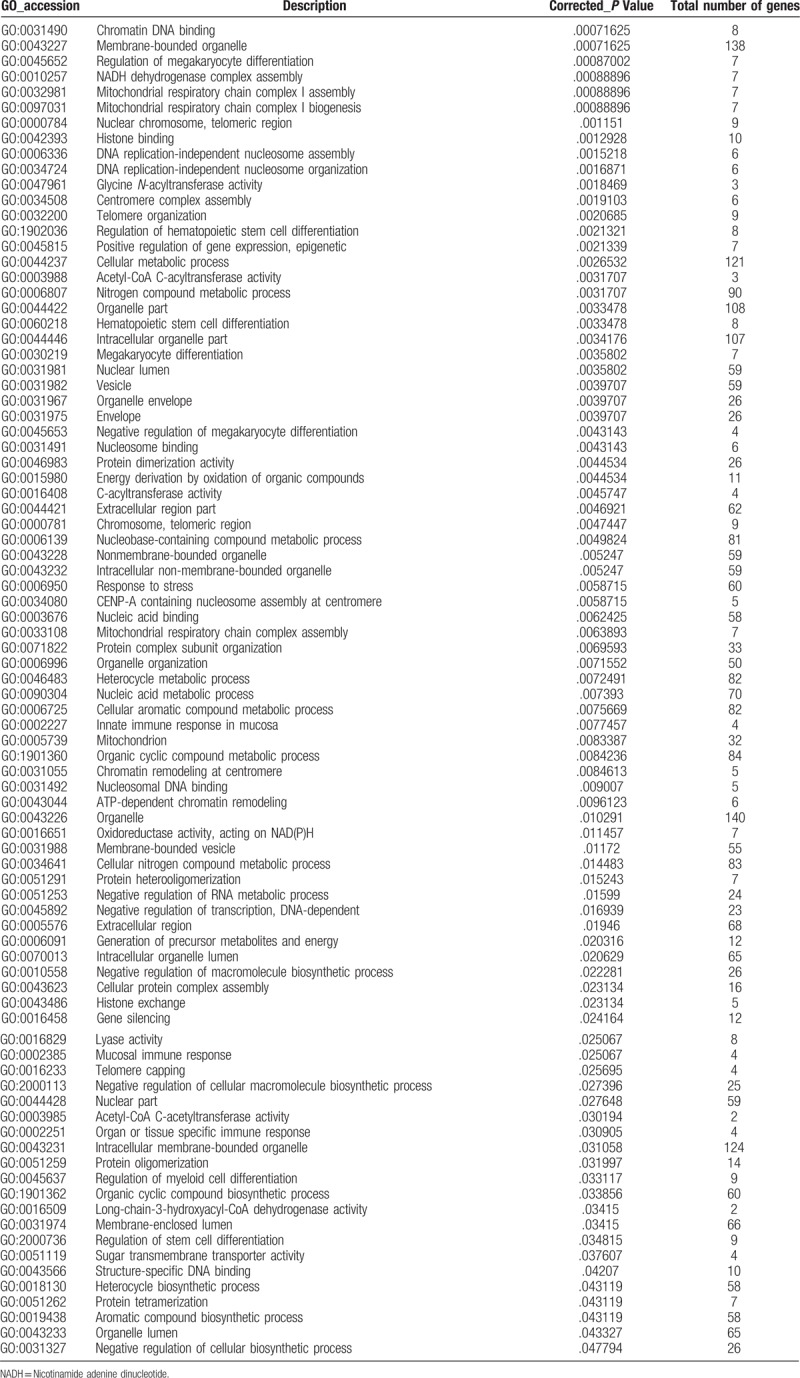
Significantly enriched gene ontology (GO) terms.

**Table 4:**
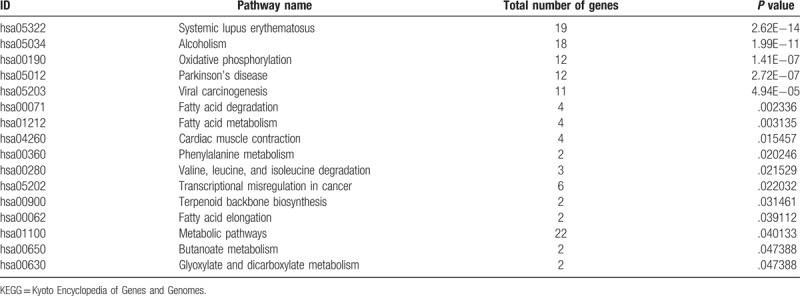
Significantly enriched KEGG pathways.

### 3.5 qRT-PCR validation of lncRNA expression

**Figure 6:**
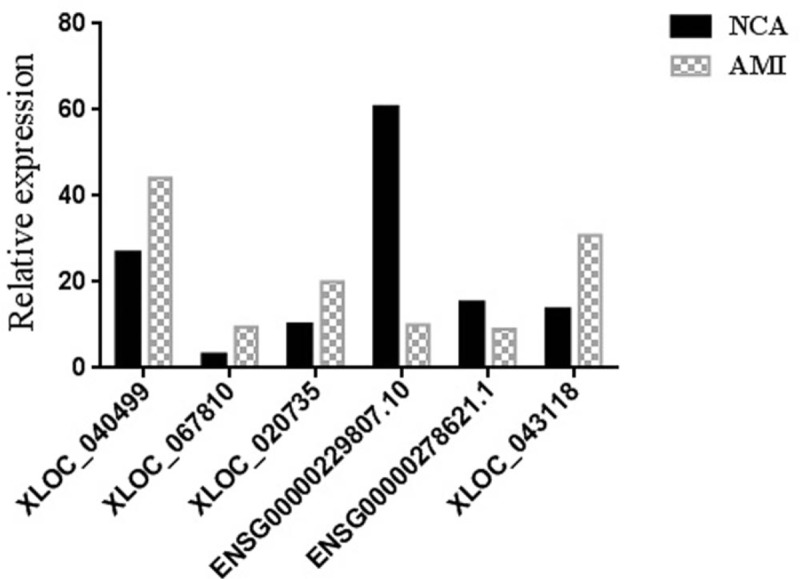
Validation of RNA-seq results by using quantitative qRT-PCR. The data are expressed as the mean ± SD (n = 30).

To validate the sequencing data of lncRNA expression level, 3 upregulated lncRNAs (XLOC_040499, XLOC_067810, and XLOC_020735) and 3 downregulated lncRNAs (ENSG00000229807.10, ENSG00000278621.1 and XLOC_043118) with only one transcript were randomly selected. We verified the differential expression of these lncRNAs from PBMCs of AMI patients (n = 30) and NCA controls (n = 30) by qRT-PCR using GAPDH as the reference gene with the 2^−ΔΔCT^ method. As shown in Figure 6, the results of qRT-PCR were consistent with the outcomes obtained from RNA sequencing analysis in the 6 differentially expressed lncRNAs of AMI patients compared with NCA controls.

## 4 Discussion

In China, deaths from cardiovascular diseases currently account for the top cause of total diseases deaths. The increasing burden of cardiovascular disease has become a major public health problem. In particular, with the aging of the population and the acceleration of urbanization, the prevalence of risk factors for cardiovascular diseases in China is significant, resulting in the continuous increase in the number of people suffering from cardiovascular diseases. The number of cardiovascular patients will continue to increase rapidly in the next 10 years.^[24–28]^

With the development of translational medicine, the role of biomarkers in cardiovascular diseases has attracted increasingly attentions. It has great application value in the early diagnosis, differential diagnosis, treatment response prediction and prognosis judgment of diseases.^[29–33]^ The ideal biomarkers should have the following characteristics, which can reflect the onset and progression of diseases or conditions, have stability, simple and easy methods to detect, small individual injuries, have high sensitivity and specificity and high economics of the economy to contribute to popularization.^[34]^ The biomarkers widely used in clinical practice including creatine kinase (CK), creatine kinase isoenzyme (CK-MB), type B natriuretic peptide (BNP) and troponin cTnI (cTnI) and so on, for they have important clinical significance in the diagnosis, treatment response and other aspects of myocardial injury, myocarditis, heart failure and other diseases.^[35–38]^ Therefore, discovering more biomarkers and giving full play to their role in precision medicine are important directions for basic research and clinical work in the future.

lncRNAs are noncoding RNAs with a length of more than 200 nt in the nucleus or cytoplasm and relatively long nucleotide chains. They have a specific and complex secondary space structure inside the molecule and can provide multiple sites for protein binding or interactions with DNA and RNA, occurring through specific and dynamic interactions, forming a complex, precise, and delicate network of gene expression and regulation. lncRNA has the characteristics of tissue specificity, cell specificity, development stage specificity, spatiotemporal specificity and disease specificity. It is widely involved in cell differentiation, metabolism and proliferation, and closely related to various diseases, including AMI. In this study, a total of 106 differentially expressed lncRNAs were discriminated in AMI patients, including 40 upregulated lncRNAs and 66 downregulated lncRNAs.

In this study, in the significantly enriched KEGG signaling pathways, systemic lupus erythematosus signaling pathway is involved in the development of immune complex deposition, vasculitis, and vascular lesions.^[39]^ Cardiac muscle contraction signaling pathway^[40]^ is associated with the onset and progression of myocardial infarction. In addition, in the significantly enriched KEGG signaling pathway, several fatty acid signaling pathways^[41,42]^ are involved in the metabolism of triglycerides, which may also be associated with the occurrence and development of myocardial infarction.

The regulation mechanism of microRNA and lncRNA in vascular injury, remodeling and aging has attracted more and more attentions. They regulate various aspects of gene expression through multiple targets, multiple pathways, such as chromatin remodeling, transcription, processing, and post-transcriptional modification.^[43–46]^ Vascular system diseases include a series of common diseases such as atherosclerosis, hypertension, myocardial infarction, stroke, pulmonary hypertension and diabetic vascular disease. Therefore, it is very important to explore the relationship between microRNA and lncRNA and vascular system diseases and clinical diagnosis. However, the understanding of the regulation mechanism of microRNA and lncRNA is still superficial, and their interaction with other regulatory mechanisms needs to be further studied. This study may help to understand that microRNA and lncRNA play an important role in maintaining the complex structure and function of blood vessels.

## 5 Conclusions

Our study used RNA sequencing to describe the comprehensive identifications and analysis of lncRNA expression profiles in AMI patients and compared them with corresponding NCA controls. The results provided differences in lncRNA expression profiles between AMI and NCA, and some of the differentially expressed lncRNAs may play a key role in various biological and pathological processes of AMI. These findings may provide useful biological information in early diagnosis and risk stratification of AMI patients. Of course, further research will be required to reveal the functional significance of abnormally expressed lncRNAs in AMI. This is one of the main research contents in our next step.

## Acknowledgments

The author would like to thank other colleagues whom were not listed in the authorship of Center for Cardiovascular Diseases, Clinical Core Laboratory and Center for Precision Medicine, Meizhou People's Hospital (Huangtang Hospital), Meizhou Hospital Affiliated to Sun Yat-sen University for their helpful comments on the manuscript. This study was supported by National Key Research and Development Program of China (Grant No.: 2017YFD0501705 to Dr PZ), National Key Research and Development Program of China (Grant No.: 2016YFD0050405 to Dr PZ), Natural Science Foundation of Guangdong Province, China (Grant No.: 2016A030307031 to Dr PZ), Medical Scientific Research Foundation of Guangdong Province, China (Grant No.: A2016306 to Dr PZ) and Key Scientific and Technological Project of Meizhou People's Hospital (Huangtang Hospital), Meizhou Hospital Affiliated to Sun Yat-sen University, Guangdong Province, China (Grant No.: MPHKSTP-20170102 to Dr PZ).

## Author contributions

Pingsen Zhao conceived and designed the experiments; Heming Wu and Pingsen Zhao recruited subjects and collected clinical data. Heming Wu conducted the laboratory testing. Zhixiong Zhong, Qifeng Zhang, Wei Zhong, Bin Li, Cunren Li, Zhidong Liu and Min Yang helped to analyze the data. Pingsen Zhao and Heming Wu prepared the manuscript.

**Conceptualization:** Pingsen Zhao.

**Data curation:** Pingsen Zhao, Zhixiong Zhong, Qifeng Zhang, Wei Zhong, Bin Li, Cunren Li, Zhidong Liu, Min Yang.

**Formal analysis:** Pingsen Zhao.

**Funding acquisition:** Pingsen Zhao.

**Investigation:** Pingsen Zhao, Heming Wu.

**Methodology:** Pingsen Zhao, Heming Wu.

**Project administration:** Pingsen Zhao.

**Resources:** Pingsen Zhao, Zhixiong Zhong, Qifeng Zhang, Wei Zhong, Bin Li, Cunren Li, Zhidong Liu, Min Yang.

**Software:** Pingsen Zhao, Heming Wu, Zhixiong Zhong.

**Supervision:** Pingsen Zhao.

**Validation:** Pingsen Zhao, Heming Wu.

**Visualization:** Pingsen Zhao.

**Writing – original draft:** Pingsen Zhao, Heming Wu.

**Writing – review & editing:** Pingsen Zhao.

## Supplementary Material

Supplemental Digital Content
