# Supplementary material for: Expression profiles of long noncoding RNAs and mRNAs in peripheral blood mononuclear cells of patients with acute myocardial infarction
Source: Medicine (Baltimore). 2018 Oct 12;97(41):e12604. doi: 10.1097/MD.0000000000012604 (PMC6203524; doi:10.1097/MD.0000000000012604)
Supplement: Supplemental Digital Content [file medi-97-e12604-s001.docx]

Table S1 Categorization of reads and basic characteristics of lncRNAs

| Sample | Raw Reads | Raw Bases | Clean Reads | Clean Bases(Gb) | Low-quality Reads Rate(%) | Adapter Related Reads Rate(%) | Ns Reads Rate(%) | Clean Q30(%) |
| --- | --- | --- | --- | --- | --- | --- | --- | --- |
| NCA-1 | 72496310 | 10.87 Gb | 65624434 | 9.84 | 9.24 | 0.23 | 0.02 | 94.39 |
| NCA-2 | 70721132 | 10.61 Gb | 64707412 | 9.71 | 8.25 | 0.22 | 0.03 | 95.07 |
| NCA-3 | 86304168 | 12.95 Gb | 75639296 | 11.35 | 12.05 | 0.29 | 0.02 | 94.14 |
| NCA-4 | 79521768 | 11.93 Gb | 73152420 | 10.97 | 7.83 | 0.18 | 0 | 95.19 |
| NCA-5 | 80209358 | 12.03 Gb | 72655618 | 10.9 | 9.12 | 0.28 | 0.02 | 94.32 |
| NCA-6 | 85820082 | 12.87 Gb | 79346492 | 11.9 | 7.32 | 0.18 | 0.04 | 95.1 |
| NCA-7 | 90964834 | 13.64 Gb | 83773232 | 12.57 | 7.65 | 0.22 | 0.04 | 95.02 |
| AMI-1 | 92472096 | 13.87 Gb | 83736836 | 12.56 | 9.21 | 0.19 | 0.05 | 94.56 |
| AMI-2 | 82211902 | 12.33 Gb | 74939050 | 11.24 | 8.59 | 0.24 | 0.02 | 94.45 |
| AMI-3 | 81417038 | 12.21 Gb | 73499784 | 11.02 | 9.43 | 0.28 | 0.02 | 94.45 |
| AMI-4 | 84510056 | 12.68 Gb | 76997902 | 11.55 | 8.68 | 0.16 | 0.05 | 94.52 |
| AMI-5 | 79991690 | 12.0 Gb | 72159492 | 10.82 | 9.59 | 0.15 | 0.05 | 94.31 |
| AMI-6 | 82561964 | 12.38 Gb | 75849696 | 11.38 | 7.95 | 0.18 | 0 | 94.38 |
| AMI-7 | 80848554 | 12.13 Gb | 73973576 | 11.1 | 8.29 | 0.21 | 0 | 94.48 |
| AMI-8 | 89874496 | 13.48 Gb | 82740772 | 12.41 | 7.84 | 0.1 | 0 | 94.6 |

Table S2 Summary of base calling and alignments

| **Sample** | **Total reads** | **Total mapped** | **Multiple mapped** | **Uniquely mapped** | **Reads map to '+'** | **Reads map to '-'** | **Non-splice reads** | **Splice reads** | **Reads mapped in proper pairs** |
| --- | --- | --- | --- | --- | --- | --- | --- | --- | --- |
| NCA-1 | 65624434 | 61846924 (94.24%) | 2087562 (3.18%) | 59759362 (91.06%) | 29930719 (45.61%) | 29828643 (45.45%) | 50766461 (77.36%) | 8992901 (13.7%) | 54287042 (82.72%) |
| NCA-2 | 64707412 | 60798357 (93.96%) | 1805409 (2.79%) | 58992948 (91.17%) | 29505057 (45.6%) | 29487891 (45.57%) | 49280223 (76.16%) | 9712725 (15.01%) | 50486876 (78.02%) |
| NCA-3 | 75639296 | 71556359 (94.6%) | 1918588 (2.54%) | 69637771 (92.07%) | 34897804 (46.14%) | 34739967 (45.93%) | 56854469 (75.17%) | 12783302 (16.9%) | 61615012 (81.46%) |
| NCA-4 | 73152420 | 68880813 (94.16%) | 2164156 (2.96%) | 66716657 (91.2%) | 33380047 (45.63%) | 33336610 (45.57%) | 55889604 (76.4%) | 10827053 (14.8%) | 59734248 (81.66%) |
| NCA-5 | 72655618 | 68602821 (94.42%) | 2128529 (2.93%) | 66474292 (91.49%) | 33312555 (45.85%) | 33161737 (45.64%) | 57768862 (79.51%) | 8705430 (11.98%) | 61525730 (84.68%) |
| NCA-6 | 79346492 | 75606510 (95.29%) | 2256014 (2.84%) | 73350496 (92.44%) | 36753935 (46.32%) | 36596561 (46.12%) | 61187455 (77.11%) | 12163041 (15.33%) | 67761372 (85.4%) |
| NCA-7 | 83773232 | 79531291 (94.94%) | 2259956 (2.7%) | 77271335 (92.24%) | 38737334 (46.24%) | 38534001 (46%) | 63705091 (76.04%) | 13566244 (16.19%) | 70953246 (84.7%) |
| AMI-1 | 83736836 | 77985333 (93.13%) | 2349407 (2.81%) | 75635926 (90.33%) | 37843178 (45.19%) | 37792748 (45.13%) | 64429936 (76.94%) | 11205990 (13.38%) | 68554376 (81.87%) |
| AMI-2 | 74939050 | 71302757 (95.15%) | 2169825 (2.9%) | 69132932 (92.25%) | 34601066 (46.17%) | 34531866 (46.08%) | 56408314 (75.27%) | 12724618 (16.98%) | 64152264 (85.61%) |
| AMI-3 | 73499784 | 68841105 (93.66%) | 2283559 (3.11%) | 66557546 (90.55%) | 33318500 (45.33%) | 33239046 (45.22%) | 57790837 (78.63%) | 8766709 (11.93%) | 61506310 (83.68%) |
| AMI-4 | 76997902 | 72544132 (94.22%) | 2431656 (3.16%) | 70112476 (91.06%) | 35098759 (45.58%) | 35013717 (45.47%) | 57014188 (74.05%) | 13098288 (17.01%) | 64509192 (83.78%) |
| AMI-5 | 72159492 | 67260163 (93.21%) | 2762900 (3.83%) | 64497263 (89.38%) | 32267891 (44.72%) | 32229372 (44.66%) | 53935383 (74.74%) | 10561880 (14.64%) | 58400784 (80.93%) |
| AMI-6 | 75849696 | 71221689 (93.9%) | 2558025 (3.37%) | 68663664 (90.53%) | 34348149 (45.28%) | 34315515 (45.24%) | 58591997 (77.25%) | 10071667 (13.28%) | 63271702 (83.42%) |
| AMI-7 | 73973576 | 69662065 (94.17%) | 2175398 (2.94%) | 67486667 (91.23%) | 33747619 (45.62%) | 33739048 (45.61%) | 58812962 (79.51%) | 8673705 (11.73%) | 61192716 (82.72%) |
| AMI-8 | 82740772 | 78594480 (94.99%) | 2605764 (3.15%) | 75988716 (91.84%) | 38009127 (45.94%) | 37979589 (45.9%) | 61887971 (74.8%) | 14100745 (17.04%) | 70339768 (85.01%) |
